# Supplementary material for: ContamLD: estimation of ancient nuclear DNA contamination using breakdown of linkage disequilibrium
Source: Genome Biol. 2020 Aug 10;21:199. doi: 10.1186/s13059-020-02111-2 (PMC7418405; doi:10.1186/s13059-020-02111-2)
Supplement: Supplementary file 1 — Additional file 1: Supplementary Figures. 10 supplementary figures showing results of additional analyses. [file 13059_2020_2111_MOESM1_ESM.docx]

**Additional file 1: Supplementary Figures**

**A) B)**


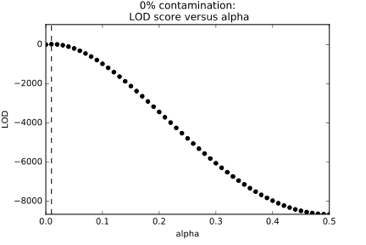

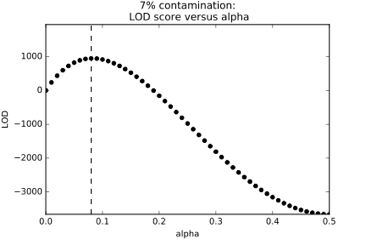


**C)**


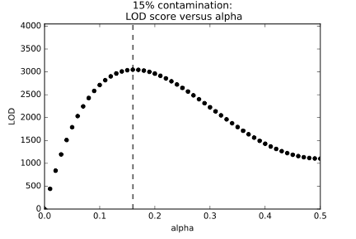


**Fig. S1. Distribution of LOD scores in simulated data.** The distribution of LOD scores is depicted for samples with **A)** 0%, **B)** 7%, and **C)** 15% simulated contamination. These data were generated as part of tests using 1000 Genomes CEU individuals as the sample and contaminant DNA and for the haplotype panel.


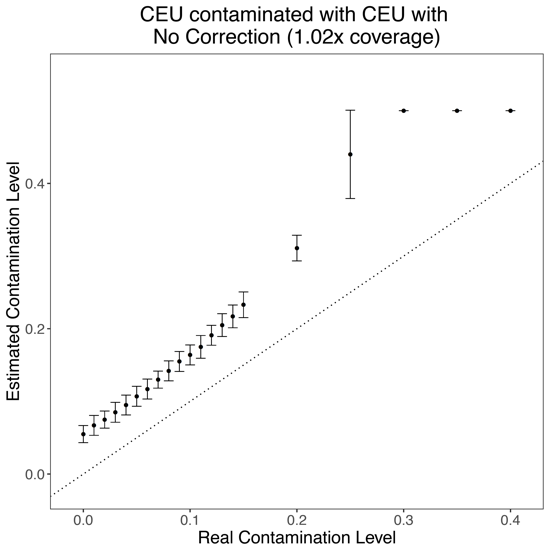


**Fig. S2. *ContamLD* estimates when the individual, contaminant, and haplotype panel are all from CEU.** *ContamLD* was run with no correction. The black dotted line is y=x, which would correspond to a perfect estimation of the contamination. Error bars are 1.96*standard error (95% confidence interval).


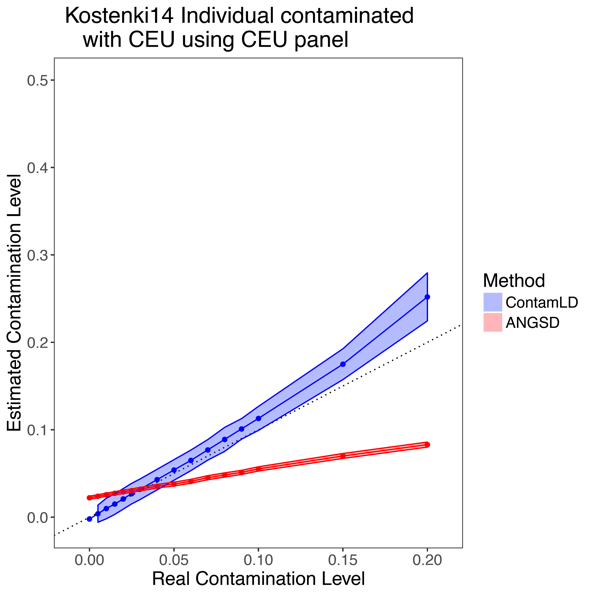


**Fig. S3. *ContamLD* estimates for Upper Paleolithic European individual after damage restricted correction (option 1).** Kostenki14 (2.81x coverage) was contaminated with CEU and analyzed using a CEU panel with *ContamLD* using damage correction and *ANGSD* [16] (Method 1). The black dotted line is y=x, which would correspond to a perfect estimation of the contamination. Error shading is 1.96*standard error (95% confidence interval).


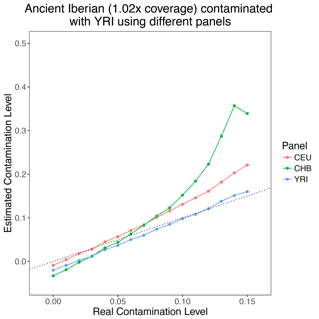

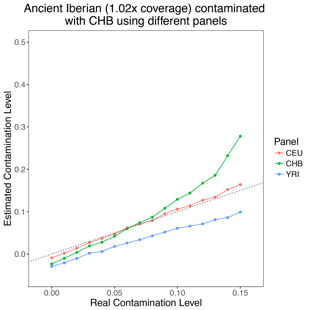


B

A

C

E

D


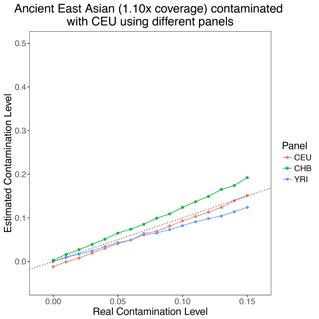

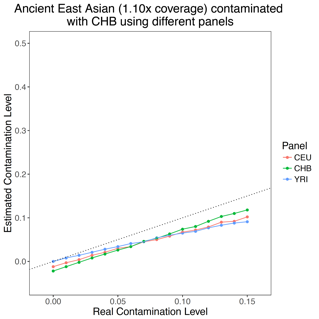

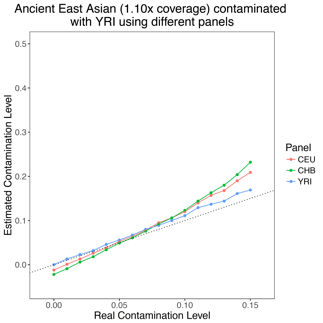


F

H

G


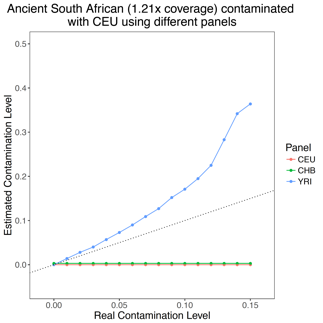

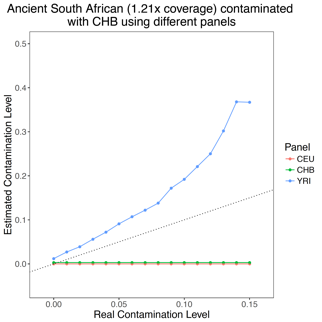

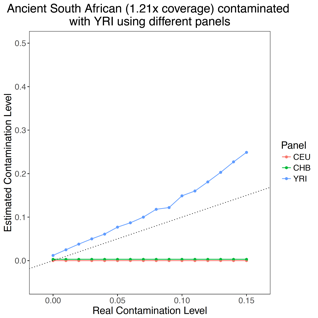


**Fig. S4. *ContamLD* estimates where the sample, contaminant, and haplotype panels have varying ancestries.** *ContamLD* was run with damage restricted correction (option 1). An ancient Iberian of 1.02x coverage (I3756) was analyzed after contamination with **A)** CHB or **B)** YRI. An ancient East Asian of 1.10x coverage (DA362.SG) is analyzed after contamination with **C)** CEU, **D)** CHB, or **E)** YRI. An ancient South African of 1.21x coverage (I9028.SG) is analyzed after contamination with **F)** CEU, **G)** CHB, or **H)** YRI. The black dotted line is y=x, which would correspond to a perfect estimation of the contamination.

**A) B)**


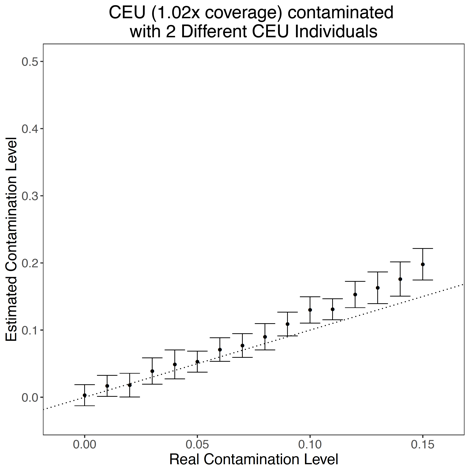

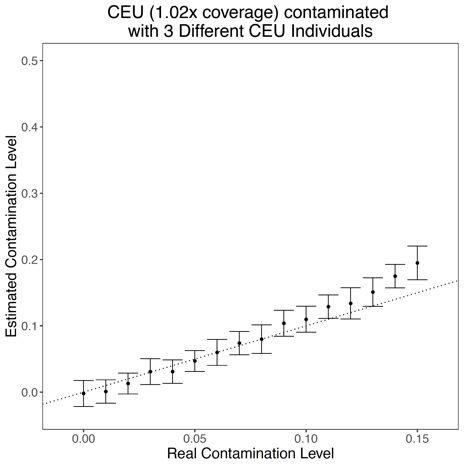


**Fig. S5. *ContamLD* estimates with CEU as the sample and multiple CEU individuals as contaminants.** *ContamLD* was run with CEU haplotype panels and damage restricted correction (option 1). A CEU individual of 1.02x coverage (from the sequence distribution of the ancient Iberian above) is contaminated with **A)** two CEU individuals or **B)** three CEU individuals. The black dotted line is y=x, which would correspond to a perfect estimation of the contamination. Error bars are 1.96*standard error (95% confidence interval).


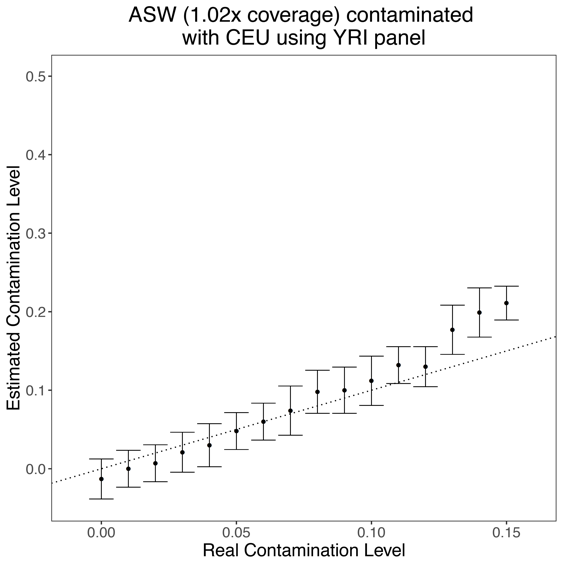


**Fig. S6. *ContamLD* estimates with an ASW (African-American) individual and YRI panel.** *ContamLD* was run with the 1240K SNP set and damage restricted correction (option 1). The black dotted line is y=x, which would correspond to a perfect estimate of the contamination. Error bars are 1.96*standard error (95% confidence interval).


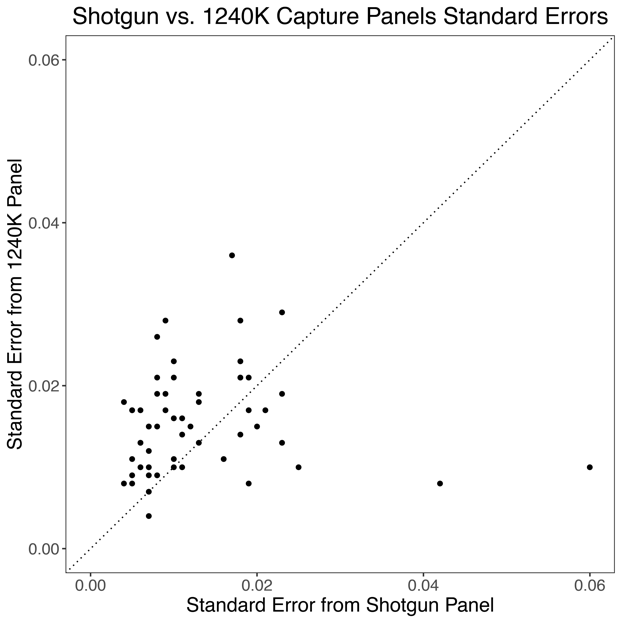


**Fig. S7. Contamination estimate standard errors of shotgun sequenced ancient individuals comparing the 1240K and shotgun panels.** Ancient shotgun sequenced individuals of 0.1-0.5x coverage from Allentoft *et al*., 2015 [31], Damgaard *et al.*, Nature 2018 [37], and Damgaard *et al*., Science 2018 [21] were analyzed with *ContamLD* damage restricted correction (option 1) using the 1240K SNP set and a shotgun panel created using all variants above 10% frequency in the 1000 Genomes dataset. This test shows that analyses with the shotgun panel generally have smaller error bars relative to those done with the 1240K panel, though it is unclear why there are two outliers with high standard errors on the shotgun panel and low standard errors on the 1240K panel. All estimates are in Table S1.


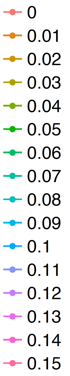

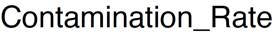

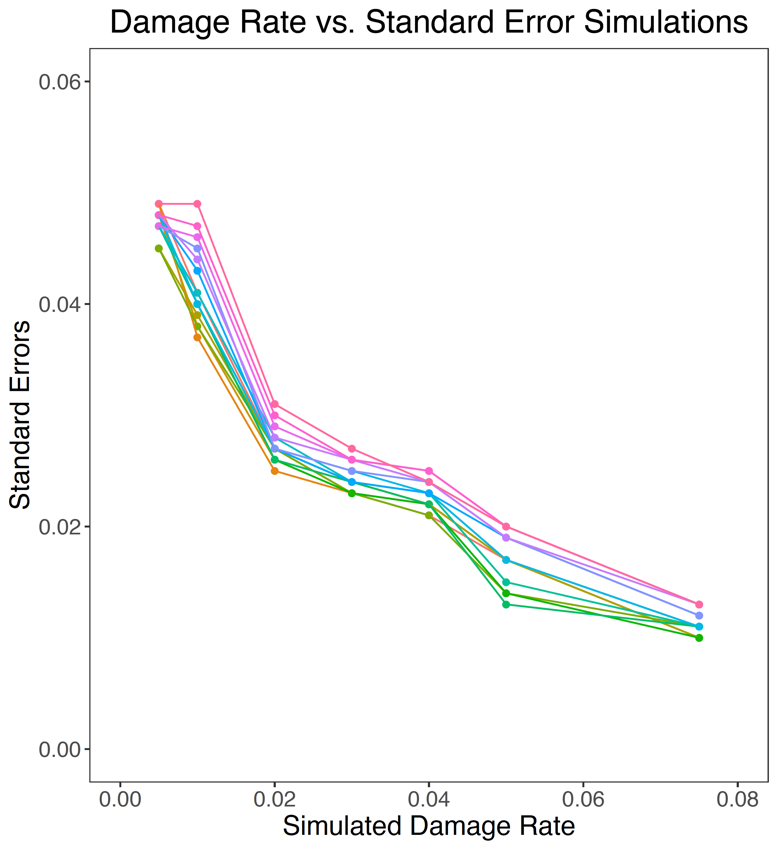


**Fig. S8. *ContamLD* standard errors from an ancient individual contaminated with another individual at different damage rates.** The target individual was a 0.5x coverage ancient West Eurasian-related individual (DA57.SG), and the contaminating individual was an ancient Iberian (I10895). *ContamLD* was run with the 1240K SNP set with the CEU panel. The damaged sequences were simulated as 0.005, 0.01, 0.02, 0.03, 0.04, 0.05, and 0.075.


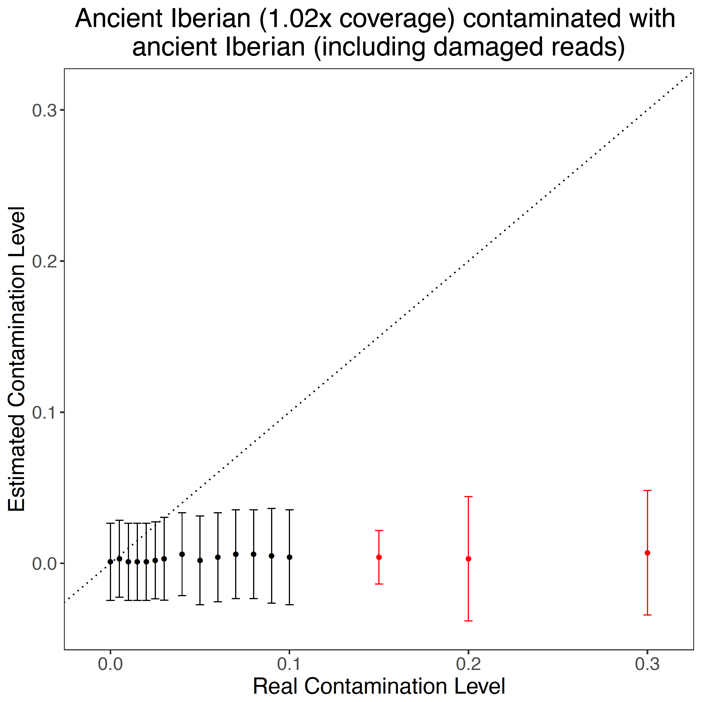


**Fig. S9. *ContamLD* estimates with an ancient individual contaminated with another ancient individual including its damaged sequences.** The target individual was an ancient Iberian (I3756) and the contaminating individual was another ancient Iberian (I10895). *ContamLD* was run with the IBS panel and 1240K SNP set using damage restricted correction (option 1). The damaged sequences were simulated as a 5% down-sampling of each respective contaminated BAM file. IBS are 1000 Genomes Project present-day Iberians from Spain. The black dotted line is y=x, which would correspond to a perfect estimation of the contamination. Error bars are 1.96*standard error (95% confidence interval). Points in red are those flagged with “Very_High_Contamination” by the software. See Table S4 for all values.

**Fig. S10.** Schematic of *ContamLD* algorithm (see text for additional details).
