# Supplementary material for: ContamLD: estimation of ancient nuclear DNA contamination using breakdown of linkage disequilibrium
Source: Genome Biol. 2020 Aug 10;21:199. doi: 10.1186/s13059-020-02111-2 (PMC7418405; doi:10.1186/s13059-020-02111-2)
Supplement: Supplementary file 6 — Additional file 6. Review history. [file 13059_2020_2111_MOESM6_ESM.docx]

**Review history**

**First round of review**

**Reviewer 1**

Nakatsuka et al. presents a very nice method for estimating contamination from ancient genomes, which is a recurrent problem that paloegenomicists face on a daily basis. With a few exceptions, existing tools are not appropriate for estimating contamination from nuclear data, which means the method has potential for being widely used in the research community. Additionally, the idea of using LD to estimate contamination is novel and appears to be quite effective.

Major concerns:

The description of the algorithm to estimate contamination is a bit sloppily written and could benefit from extensive re-writing. Perhaps it would also help to have an accompanying schematic describing some of the concepts (e.g. the two chromosomes being sampled from the same parent vs. the background). For example, in line 542 it says that p_tilde can be estimated from an ML estimator but the equation describing the estimator has no mention of p_tilde. The description of the E and M steps could be better motivated (what is n1 the expectation of? what parameter are you maximizing in the M step?, etc). In line 521, you mention the "distribution underlying the haplotypes", what do you mean by this? the distribution of the joint frequencies of the haplotypes? Please re-write this so that a person who is not familiar with the method would be able to follow the math.

To give a better understanding to the reader of performance across different panels and to the specific effects of differences in ancestry between the contaminant and the test panel, I would expand figure 2.B into multiple figures and add some more scenarios. It's a bit unclear at the moment why you choose the particular combinations of ancient_genome+contaminant+test_panel (e.g. why are you only testing CEU as contaminant and YRI as panel when working with the ancient South African) that you are including in this figure. I think it would be best if you separated this figure into 3 different figures (one for each ancient genome from different corners of the world) and plotted for each ancient genome the same consistent combinations of panels and contaminants, including combinations that seem to be missing from this plot (e.g. EastAsian_CEUcontam_YRIpanel).

You say that "scripts and simulations are available upon request". Please don't do this. Upload them to a GitHub page or some other online repository so that they are publicly available without someone having to request them to you.

Could you have a couple of plots showing estimates of bias and error (using simulated data) as a function of coverage? You could also do this as a function of other parameters, like the amount of damage or the distance between the contaminant panel and the ancient genome, etc.  It would be nice for potential users to see how low in coverage they can go (for both the shotgun and 1240k approaches) or when they could expect the method not to work a priori.

Minor concerns:

Line 188: you're missing the word "could" before "be"

In the introduction, you could also cite Moreno-Mayar et al. 2020 (https://academic.oup.com/bioinformatics/article/36/3/828/5554699), and, if possible, see if you can compare their method with your method (obviously just for males).

In the introduction, I would also mention Philip Johnson's method from the Neanderthal genome paper. My work in DICE built heavily on his approach.

In line 605, are you doing this over a grid of alpha parameters? If so, how fine-scaled is the grid?

Signed: Fernando Racimo

**Reviewer 2**

Nakatsuka describe a program, contamLD, designed to address a specific and important question in ancient DNA data analysis: are data from a specific sample contaminated with data from another individual. They leverage linkage information, available from 1000 Genomes data, to estimate a level of contamination consistent with the alleles that are being observed. The core insight is that uncontaminated samples should produce allelic observations that are predicted by other allelic observations and the linkage known from the panel. Contaminated samples, on the other hand, will not since allelic observations will be from a randomly drawn chromosome unrelated to other allelic observations in the data.

The method description is clear. Their experiments and analyses are well-designed and cover the range of possible samples, reference panels, and contamination scenarios. Their correction step is practical and solves the problem of not knowing what the baseline LD should be. In summary, the manuscript is well-done and should be published. Below are several points for possible improvement of the manuscript. None of these are compelling reasons not to publish the paper. Rather, they are offered in the hopes that consideration can further improve an already good paper.

1. The authors write that they provide tools for choosing the best reference panel but do not describe how this is done. I assume it's the usual admixtools and PCA plots. I clear description would be useful.
2. I would be interested in see how the proportion of damaged sequences affects the contamination correction results. The extent of cytosine deamination can vary wildly between samples. I'd guess that the variance in their corrected contamination estimate would increase when fewer sequences are damaged.
3. The authors may wish to consider the possibility that remains collected from the same burial site belong to related individuals. How would their contamination estimate do in the case of contamination from a related individual?
4. The contamination estimate confidence intervals are estimated by jackknife. But the specific details are missing - perhaps this is easily available in the code. I have not checked. This is an interesting point because LD patterns may change regionally within a sample due to short or long ROH, as pointed out by the authors.
5. A similar approach (LD based analysis of low-coverage sequence data) aimed at a different problem, forensic identification, was introduced by Vohr et al., BMC Genomics (2015). The authors may consider contrasting their method and goals.
6. Line 188 type "…this inbreed effect [could?] be corrected…"
7. Figure 3 appears to show contamination estimates and confidence intervals less than 0%. It's not clear how this is possible or how it should be interpreted.
8. Line 288: The wording of the sentence starting here is unclear. Consider this alternative: We used data from male individuals selected due to very low X chromosome contamination estimates from ANGSD.
9. Line 434 "not unbiased" is probably meant to read "not biased"
10. The ~1 million or ~5 million SNPs that are chosen for analysis are likely those that are part of the normal analysis procedure from this lab. These sites were likely LD pruned or otherwise selected to provide maximally independent data. This procedure, leveraging LD between sites, is at a cross-purpose. The authors may wish to comment on this. Specifically, the idea of expanding SNP sites to include some pairs that maximize LD to increase the power of this contamination estimation approach could be considered.

**Response of authors**

Reviewer reports:

Reviewer #1: Nakatsuka et al. presents a very nice method for estimating contamination from

ancient genomes, which is a recurrent problem that paloegenomicists face on a daily basis. With

a few exceptions, existing tools are not appropriate for estimating contamination from nuclear

data, which means the method has potential for being widely used in the research community.

Additionally, the idea of using LD to estimate contamination is novel and appears to be quite

effective.

Major concerns:

The description of the algorithm to estimate contamination is a bit sloppily written and could

benefit from extensive re-writing. Perhaps it would also help to have an accompanying schematic

describing some of the concepts (e.g. the two chromosomes being sampled from the same parent

vs. the background). For example, in line 542 it says that p_tilde can be estimated from an ML

estimator but the equation describing the estimator has no mention of p_tilde. The description of

the E and M steps could be better motivated (what is n1 the expectation of? what parameter are

you maximizing in the M step?, etc). In line 521, you mention the "distribution underlying the

haplotypes", what do you mean by this? the distribution of the joint frequencies of the

haplotypes? Please re-write this so that a person who is not familiar with the method would be

able to follow the math.

**We apologize for the lack of clarity in our previous write-up. We have now cleaned this up,**

**changed some of the notation, and added more detail as well as a schematic and hope that**

**this improves clarity.**

To give a better understanding to the reader of performance across different panels and to the

specific effects of differences in ancestry between the contaminant and the test panel, I would

expand figure 2.B into multiple figures and add some more scenarios. It's a bit unclear at the

moment why you choose the particular combinations of

ancient_genome+contaminant+test_panel (e.g. why are you only testing CEU as contaminant

and YRI as panel when working with the ancient South African) that you are including in this

figure. I think it would be best if you separated this figure into 3 different figures (one for each

ancient genome from different corners of the world) and plotted for each ancient genome the

same consistent combinations of panels and contaminants, including combinations that seem to

be missing from this plot (e.g. EastAsian_CEUcontam_YRIpanel).

**This has now been done and put in Supplementary Figure 4. We left Figures 2A and 2B,**

**because those show the salient points about the effect of the panels and contaminants, but**

**the findings are general and we now point to the new analyses to document this.**

You say that "scripts and simulations are available upon request". Please don't do this. Upload

them to a GitHub page or some other online repository so that they are publicly available without

someone having to request them to you.

**We have now uploaded these to the GitHub page.**

Could you have a couple of plots showing estimates of bias and error (using simulated data) as a

function of coverage? You could also do this as a function of other parameters, like the amount

of damage or the distance between the contaminant panel and the ancient genome, etc. It would

be nice for potential users to see how low in coverage they can go (for both the shotgun and

1240k approaches) or when they could expect the method not to work a priori.

**We believe we have addressed this with Figure 3 showing no bias but increasing standard**

**error as the coverage goes down. However, we also performed additional simulations to**

**address this with a 1.0x coverage ancient West Eurasian ancestry individual (DA57.SG, an**

**ancient Krgyzstanian individual of 1.0x coverage) (Damgaard *et* al., 2018, Nature)**

**contaminated with I10895, a West Eurasian ancestry individual (for 1240K SNP set**

**analyses), or a present-day whole-genome sequenced French individual (Mallick *et al*., 2016**

**Nature) (for shotgun SNP set analyses). To simulate different coverages, we performed**

**down-sampling of the DA57.SG individual to 0.1, 0.2, 0.3, 0.4, 0.5, 0.75, and 1x coverages,**

**and to simulate different damage rates, we varied the damage rate to the proportions (0.01,**

**0.02, 0.03, 0.04, 0.05, 0.075) by setting the amount of “damaged” sequences to be those**

**proportions and analyzed DA57.SG when it was down-sampled to 0.25x coverage. We used**

***ContamLD* with both the 1240K and shotgun SNP sets and the CEU, CHB, and YRI panels.**

**We found that for the CEU panel, the 1240K SNP set performed well at coverages of at**

**least 0.3x, while shotgun SNP set performed well at coverages of at least 0.2x (Reviewer**

**Figure 1A-F)). With the CHB and YRI panels the performance was poor at all levels of**

**coverage (Reviewer Figure 1G-R) as predicted from Figure 2.**

**For the damage rate simulation (Reviewer Figure 2 and Supplementary Figure 8) we show**

**below that the power of *ContamLD* increases with increased number of damaged sequences**

**(most likely because the limiting factor for the power is usually the estimates based on**

**damaged reads), though the effect was not as large as we expected. *ContamLD* worked well**

**for the 0.25x coverage simulation at all damage rates except 0.01, which is lower than the**

**damage rate of most ancient DNA samples even after partial UDG treatment. This also**

**means that libraries not treated with UDG will have increased power relative to libraries**

**from the same extracts not treated with UDG as discussed in the text.**

**However, after much discussion we decided not to put these figures in the manuscript,**

**because we believe for the analysis to be rigorous we would need to perform it for many**

**dozens of individuals (~50-100 ideally) to determine when the contamination estimates are**

**significantly deviated (>2 SEs) from the true contamination levels. The analyses presented**

**below were only for a single individual, so it is unclear whether this holds in general (for**

**example, even for the lowest coverages with the CEU panel the *ContamLD* estimates were**

**within 2 SEs of the true contamination level showing there was not necessarily a systematic**

**bias in the results at that coverage, though the standard errors were higher at the lower**

**coverages). We were unfortunately unable to perform the analysis for the necessary**

**number of individuals due to the enormous computational requirements it would involve.**

**Nevertheless, we believe the results previously in the manuscript show the relevant findings**

**present in these analyses. On a practical level, as coverage becomes low and standard**

**errors increase, the estimates naturally become less reliable (e.g. if the standard errors are**

**over 3 percent, then the confidence interval for the estimates could be beyond levels the**

**user is willing to accept).**

**Reviewer Figure 1. *ContamLD* estimates with an ancient individual contaminated with another ancient individual at different coverages.** The target individual was ancient individual DA57.SG (a Saka culture individual from the Tian Shan mountains of mostly West Eurasian-related ancestry), and the contaminating individual was an ancient Iberian (I10895) for the 1240K SNP set and a present day French individual for the shotgun SNP set. The coverages were simulated as **(A)** 0.1x, **(B)** 0.2x, **(C)** 0.3x, **(D)** 0.4x, **(E)** 0.5x, **(F)** 0.75x, and **(G)** 1x using the CEU panel, and the same coverages using the **(G-L)** CHB and **(M-R)** YRI panels.

**Reviewer Figure 2. *ContamLD* estimates with an ancient individual contaminated with another ancient individual at different damage rates.** The target

individual was a 0.25x coverage ancient West Eurasian-related individual (DA57.SG), and the contaminating individual was an ancient Iberian (I10895). *ContamLD* was run with the 1240K SNP set with the CEU panel. The damaged sequences were simulated as **(A)** 0.01, **(B)** 0.02, **(C)** 0.03, **(D)** 0.04, **(E)** 0.05, and **(F)** 0.075.

Minor concerns:

Line 188: you're missing the word "could" before "be"

**This has been corrected.**

In the introduction, you could also cite Moreno-Mayar et al. 2020

(https://academic.oup.com/bioinformatics/article/36/3/828/5554699), and, if possible, see if you

can compare their method with your method (obviously just for males).

**Thanks for drawing our attention to this method, which we now cite in the revised**

**introduction. On reflection we decided not to add an analysis comparing the results of their**

**method to ours, as in our experience ANGSD works well at the coverages we used for our**

**validation analyses. The new Moreno-Mayar et al. 2020 method—while an advance relative**

**to ANGSD—like ANGSD still has the limitation that it only works for males. The primary**

**advance of our method is to be able to measure contamination in ancient female**

**individuals, and we make no claim of outperforming ANGSD (the most commonly tool for**

**assessing nuclear DNA contamination) in males, so we did not feel it was critical for the**

**present study to add this comparison.**

In the introduction, I would also mention Philip Johnson's method from the Neanderthal genome

paper. My work in DICE built heavily on his approach.

**We have now mentioned this method in the introduction.**

In line 605, are you doing this over a grid of alpha parameters? If so, how fine-scaled is the grid?

**We have now specified that this is done over a grid of** *α*′ **scaled by intervals of 0.0001.**

Signed: Fernando Racimo

Reviewer #2: Nakatsuka describe a program, contamLD, designed to address a specific and

important question in ancient DNA data analysis: are data from a specific sample contaminated

with data from another individual. They leverage linkage information, available from 1000

Genomes data, to estimate a level of contamination consistent with the alleles that are being

observed. The core insight is that uncontaminated samples should produce allelic observations

that are predicted by other allelic observations and the linkage known from the panel.

Contaminated samples, on the other hand, will not since allelic observations will be from a

randomly drawn chromosome unrelated to other allelic observations in the data.

The method description is clear. Their experiments and analyses are well-designed and cover the

range of possible samples, reference panels, and contamination scenarios. Their correction step is

practical and solves the problem of not knowing what the baseline LD should be. In summary,

the manuscript is well-done and should be published. Below are several points for possible

improvement of the manuscript. None of these are compelling reasons not to publish the paper.

Rather, they are offered in the hopes that consideration can further improve an already good

paper.

1. The authors write that they provide tools for choosing the best reference panel but do not

describe how this is done. I assume it's the usual admixtools and PCA plots. I clear description

would be useful.

**In the revised manuscript we now specify that this is done via outgroup-*f3* statistics (shared**

**genetic drift). As we discuss, the user can also usually guess the reference panel based on**

**the continental ancestry of the individual with good results.**

2. I would be interested in see how the proportion of damaged sequences affects the

contamination correction results. The extent of cytosine deamination can vary wildly between

samples. I'd guess that the variance in their corrected contamination estimate would increase

when fewer sequences are damaged.

**This is an important suggestion. We performed additional simulations to address this with**

**an ancient West Eurasian ancestry individual (DA57.SG, an ancient Krgyzstanian**

**individual) (Damgaard *et* al., 2018, Nature) down-sampled to 0.25x coverage and**

**contaminated with I10895, a West Eurasian ancestry individual. We varied the damage**

**rate to the proportions (0.01, 0.02, 0.03, 0.04, 0.05, 0.075) by setting the amount of**

**“damaged” sequences to be those proportions. We show below and in Supplementary**

**Figure 8 that, as the reviewer suggests, the power of *ContamLD* increases with increased**

**number of damaged sequences (most likely because the limiting factor for the power is**

**usually the estimates based on damaged reads), though the effect was not as large as we**

**expected. *ContamLD* worked well for the 0.25x coverage simulation at all damage rates**

**except 0.01, which is lower than the damage rate of most ancient DNA samples even after**

**partial UDG treatment. This also means that libraries not treated with UDG will have**

**increased power relative to libraries from the same extracts not treated with UDG.**

**However, after much discussion we decided not to put Reviewer Figure 2 in the**

**manuscript, because we believe for the analysis to be rigorous we would need to perform it**

**for many dozens of individuals (~50-100 ideally) to determine when the contamination**

**estimates are significantly deviated (>2 SEs) from the true contamination levels. The**

**analysis presented below is only for a single individual, so it is unclear whether this holds in**

**general. We were unfortunately unable to perform the analysis for the necessary number of**

**individuals due to the enormous computational requirements it would involve.**

**Nevertheless, as we discuss in the text, the data from partially UDG-treated libraries**

**subjected to in-solution enrichment that we focus on in our own laboratory and that is the**

**basis for much of our empirical testing in the paper represents a fairly bad scenario for**

**ContamLD, which works best for libraries that are not UDG-treated and that are shotgun**

**sequenced. The fact that the method works as well as it does in this scenario thus provides**

**an encouraging baseline.**

**Second round of review**

**Reviewer 1**

The authors have addressed all my concerns.
